# Effects of Dapagliflozin on Symptoms, Function, and Quality of Life in Patients With Heart Failure and Reduced Ejection Fraction: Results From the DAPA-HF Trial

**DOI:** 10.1161/CIRCULATIONAHA.119.044138

**Published:** 2019-11-17

**Authors:** Mikhail N. Kosiborod, Pardeep S. Jhund, Kieran F. Docherty, Mirta Diez, Mark C. Petrie, Subodh Verma, Jose C. Nicolau, Béla Merkely, Masafumi Kitakaze, David L. DeMets, Silvio E. Inzucchi, Lars Køber, Felipe A. Martinez, Piotr Ponikowski, Marc S. Sabatine, Scott D. Solomon, Olof Bengtsson, Daniel Lindholm, Anna Niklasson, Mikaela Sjöstrand, Anna Maria Langkilde, John J.V. McMurray

**Affiliations:** 1 Saint Luke’s Mid America Heart Institute, University of Missouri-Kansas City (M.N.K.).; 2 The George Institute for Global Health and University of New South Wales, Sydney, Australia (M.N.K).; 3 British Heart Foundation Cardiovascular Research Centre, University of Glasgow, UK (P.S.J., K.F.D., M.C.P., J.J.V.M.).; 4 Division of Cardiology, Instituto Cardiovascular de Buenos Aires, Argentina (M.D.).; 5 St Michael’s Hospital, University of Toronto, Canada (S.V.).; 6 Instituto do Coracao (InCor), Hospital das Clínicas Faculdade de Medicina, Universidade de São Paulo, Brazil (J.C.N.).; 7 Heart and Vascular Center, Semmelweis University, Budapest, Hungary (B.M.).; 8 Department of Clinical Medicine and Development, National Cerebral and Cardiovascular Center, Suita Osaka, Japan (M.K.).; 9 Department of Biostatistics and Medical Informatics, University of Wisconsin, Madison (D.L.D.).; 10 Section of Endocrinology, Yale School of Medicine, New Haven, CT (S.E.I.).; 11 Rigshospitalet, Department of Cardiology, University of Copenhagen, Denmark (L.K.).; 12 Universidad Nacional de Córdoba, Argentina (F.A.M.).; 13 Center for Heart Diseases, University Hospital, Wroclaw Medical University, Poland (P.P.).; 14 Division of Cardiovascular Medicine, Brigham and Women’s Hospital, Boston, MA (M.S.S., S.D.S.).; 15 Late Stage Development, Cardiovascular, Renal, and Metabolism, BioPharmaceuticals R&D, AstraZeneca, Gothenburg, Sweden (O.B., D.L., A.N., M.S., A.M.L.).

**Keywords:** health status, heart failure, outcome, sodium-glucose transporter 2 inhibitors

## Abstract

**Background:**

Goals of management in patients with heart failure and reduced ejection fraction include reducing death and hospitalizations, and improving health status (symptoms, physical function, and quality of life). In the DAPA-HF trial (Dapagliflozin and Prevention of Adverse-Outcomes in Heart Failure), sodium–glucose cotransporter-2 inhibitor, dapagliflozin, reduced death and hospitalizations, and improved symptoms in patients with heart failure and reduced ejection fraction. In this analysis, we examine the effects of dapagliflozin on a broad range of health status outcomes, using the Kansas City Cardiomyopathy Questionnaire (KCCQ).

**Methods:**

KCCQ was evaluated at randomization, 4 and 8 months. Patients were divided by baseline KCCQ total symptom score (TSS); Cox proportional hazards models examined the effects of dapagliflozin on clinical events across these subgroups. We also evaluated the effects of dapagliflozin on KCCQ-TSS, clinical summary score, and overall summary score. Responder analyses were performed to compare proportions of dapagliflozin versus placebo-treated patients with clinically meaningful changes in KCCQ at 8 months.

**Results:**

A total of 4443 patients had available KCCQ at baseline (median KCCQ-TSS, 77.1 [interquartile range, 58.3–91.7]). The effects of dapagliflozin vs placebo on reducing cardiovascular death or worsening heart failure were consistent across the range of KCCQ-TSS (lowest to highest tertile: hazard ratio, 0.70 [95% CI, 0.57–0.86]; hazard ratio, 0.77 [95% CI, 0.61–0.98]; hazard ratio, 0.62 [95% CI, 0.46–0.83]; *P* for heterogeneity=0.52). Patients treated with dapagliflozin had greater improvement in mean KCCQ-TSS, clinical summary score, and overall summary score at 8 months (2.8, 2.5 and 2.3 points higher versus placebo; *P*<0.0001 for all). Fewer patients treated with dapagliflozin had a deterioration in KCCQ-TSS (odds ratio, 0.84 [95% CI, 0.78–0.90]; *P*<0.0001); and more patients had at least small, moderate, and large improvements (odds ratio, 1.15 [95% CI, 1.08–1.23]; odds ratio, 1.15 [95% CI, 1.08–1.22]; odds ratio, 1.14 [95% CI, 1.07–1.22]; number needed to treat=14, 15, and 18, respectively; *P*<0.0001 for all; results consistent for KCCQ clinical summary score and overall summary score).

**Conclusions:**

Dapagliflozin reduced cardiovascular death and worsening heart failure across the range of baseline KCCQ, and improved symptoms, physical function, and quality of life in patients with heart failure and reduced ejection fraction. Furthermore, dapagliflozin increased the proportion of patients experiencing at least small, moderate, and large improvements in health status; these effects were clinically important.

**Clinical Trial Registration:**

URL: https://www.clinicaltrials.gov. Unique identifier: NCT03036124.

## Abstract 2

Supplemental Digital Content is available in the text.

**Clinical Perspective**

**What Is New?**

- Dapagliflozin improved cardiovascular death or worsening heart failure in patients with heart failure and reduced ejection fraction regardless of the level of symptomatic impairment at baseline.
- Dapagliflozin improved symptom burden, physical function, and quality of life in patients with heart failure and reduced ejection fraction; these effects were sustained and amplified over time.
- Dapagliflozin significantly increased the proportion of patients experiencing at least small, moderate, and large improvements in health status; these effects were clinically important.

**What Are the Clinical Implications?**

- The beneficial effects of dapagliflozin on heart failure outcomes are independent of the health status impairment at baseline.
- Our findings indicate that dapagliflozin significantly improves heart failure–related health status (symptoms, physical function, and quality of life), as measured by the Kansas City Cardiomyopathy Questionnaire, with the benefits emerging early and being sustained long-term.

**Editorial, see p 112**

Patients with heart failure (HF) and reduced ejection fraction (HFrEF) are at high risk of disease progression, resulting in clinical deterioration, repeat hospitalizations, and death.^1^ Importantly, they also experience a high burden of debilitating symptoms, which impact their daily function and quality of life. Of note, some treatments for HFrEF (such as *β*-blockers) that have a favorable effect on death and hospitalizations do not improve health status,^2^ highlighting the high unmet need for additional efficacious therapies that not only improve clinical events but also reduce symptom burden and physical limitations, and improve the quality of life. In fact, improving patients’ health status (which includes symptom burden, physical limitations, and quality of life) is a key goal of HF management, increasingly recognized by the practice guidelines,^3,4^ and acknowledged by regulators as an important outcome.^5^

In the placebo-controlled DAPA-HF trial (Dapagliflozin and Prevention of Adverse-Outcomes in Heart Failure) trial, the sodium–glucose cotransporter-2 inhibitor, dapagliflozin, added to other guideline-recommended therapies, reduced the risk of mortality and HF hospitalization, and improved symptoms in 4744 patients with HFrEF.^6^ In the current analysis, we sought to address the following two objectives: (1) to evaluate whether the effects of dapagliflozin on clinical outcomes in the DAPA-HF trial varied according to the degree of symptomatic impairment at baseline; and (2) to examine the effects of dapagliflozin on the broad range of health status outcomes, as measured by the various domains of the Kansas City Cardiomyopathy Questionnaire (KCCQ)—a validated, self-administered instrument that quantifies HF-related symptoms, function, and quality of life.

## Methods

Data underlying the findings described in this manuscript may be obtained in accordance with AstraZeneca’s data sharing policy.^7^ DAPA-HF was a randomized, double-blind, controlled trial in patients with HFrEF, which evaluated the efficacy and safety of dapagliflozin 10 mg once daily, compared with matching placebo, added to standard care. The design, baseline characteristics, and primary results of the trial have been published.^8^ The Ethics Committee of each of the 410 participating institutions (in 20 countries) approved the protocol, and all patients gave written informed consent. The corresponding author had full access to all of the trial data and takes responsibility for its integrity and the data analysis.

### Study Patients

Men and women aged ≥18 years with HF were eligible if they were in New York Heart Association (NYHA) functional class ≥II, had a left ventricular ejection fraction ≤40%, and were optimally treated with pharmacological and device therapy for HF. Participants were also required to have a NT-proBNP (N-terminal pro-B-type natriuretic peptide) concentration ≥600 pg/mL (≥400 pg/mL if hospitalized for HF within the previous 12 months). Patients with atrial fibrillation or atrial flutter were required to have a NT-proBNP level ≥900 pg/mL, irrespective of history of HF hospitalization. Key exclusion criteria included the following: symptoms of hypotension or systolic blood pressure <95 mm Hg, estimated glomerular filtration rate <30 mL/min per 1.73 m^2^ (or rapidly declining renal function), type 1 diabetes mellitus, and other conditions likely to prevent patient participation in the trial or greatly limit life expectancy. A full list of exclusion criteria is provided in the design manuscript.^9^

### Study Procedures

After the provision of informed consent, visit 1 started a 14-day screening period during which the trial inclusion and exclusion criteria were checked, and baseline information was collected. Visit 2 was the randomization visit, and randomization was stratified based on diagnosis of type 2 diabetes mellitus (defined as an established diagnosis or a glycated hemoglobin level of ≥6.5% [≥48 mmol per mole]). After randomization, follow-up visits took place at 14 and 60 days, and then at 120, 240, and 360 days and every 4 months thereafter. The visit early after randomization (14 days) was included to check renal function and blood pressure (as well as for symptoms of hypotension); this visit also allowed for adjustment of background diuretic or other nonessential therapies. Dose reduction (to 5 mg daily of dapagliflozin or placebo) or temporary discontinuation of study drug was to be considered in case of an acute unexpected decline in estimated glomerular filtration rate, volume depletion, or hypotension (or to avoid these conditions); however, dose up-titration (or reinitiation) was encouraged thereafter in all cases, where possible.

### Clinical Outcomes

The primary outcome in the DAPA-HF trial was the composite of an episode of worsening HF (HF hospitalization or urgent HF visit) or cardiovascular death, whichever occurred first. Additional clinical outcomes assessed in the current study were the occurrence of HF hospitalization or cardiovascular death; worsening HF events (HF hospitalizations or urgent HF visits), hospitalization for HF, cardiovascular death, and all-cause death.

### Kansas City Cardiomyopathy Questionnaire

The KCCQ was completed electronically by patients, without assistance by site study staff (as validated), and evaluated at randomization, 4 months, and 8 months. The KCCQ is a 23-item, self-administered disease-specific instrument that quantifies symptoms (frequency, severity, and recent change), physical function, quality of life, and social function over the previous 2 weeks. In the KCCQ, the total symptom score (TSS) quantifies the symptom frequency and severity, KCCQ clinical summary score (CSS) includes the physical function and symptoms domains, and KCCQ overall summary score (OSS) is derived from total symptom score, physical function, quality of life, and social function. For each domain, the validity, reproducibility, responsiveness, and interpretability have been independently established. Scores are transformed to a range of 0 to 100, in which higher scores reflect better health status.^10^

### Statistical Analysis

In the present post hoc analysis, patients were divided into 3 subgroups, based on the tertiles of baseline KCCQ-TSS (which was the KCCQ domain prespecified as the secondary end point): (i) ≤65.6, (ii) 65.7 to 87.5, and (iii) >87.5 points. Baseline characteristics were summarized as means and SDs, medians, and interquartile ranges, or percentages. A Cuzick extension of the Wilcoxon test for trend was used to compare trends across categories of KCCQ.^11^ The rates of cardiovascular death and worsening HF across the tertiles of KCCQ-TSS (regardless of treatment allocation) were calculated and compared using Kaplan-Meier estimates.

To compare the effects of dapagliflozin versus placebo on clinical outcomes across the KCCQ-TSS tertiles, we evaluated time-to-event data with the use of Kaplan-Meier estimates and used Cox proportional-hazards models, stratified according to diabetes mellitus status, with a history of HF hospitalization and treatment-group assignment as fixed-effect factors to calculate hazard ratios (HR), 95% CIs, and two-sided *P* values.

We analyzed the differences between treatment groups in mean KCCQ-TSS, CSS, and OSS at 4 months and 8 months in surviving patients, using a mixed model for repeated measurements and estimated the least-squares mean differences between treatment groups adjusted for baseline KCCQ values. We conducted responder analyses examining proportions of patients with a deterioration, and clinically important improvements in KCCQ at 8 months. We used established, clinically meaningful thresholds for KCCQ (≥5 point [at least small], ≥10 point [moderate], and ≥15 point [large] change) for all responder analyses across the KCCQ domains.^12^ The proportion of responders was compared between those treated with dapagliflozin vs placebo using multiple imputation to account for missing KCCQ values (see below). Odds ratios (ORs) to estimate differences between treatment groups, and their corresponding 95% CI and 2-sided *P* values were estimated from logistic regression models (which included treatment group, stratification variable (type 2 diabetes mellitus at randomization), and baseline KCCQ values); the models used imputed data accounting for missing KCCQ values, and estimates were combined using Rubin’s rules. Missing data were imputed using a missing at random assumption and a predictive mean matching multiple imputation model, and a method of Fully Conditional Specification as implemented in the SAS Procedure MI (Fully Conditional Specification [FCS] statement). The imputation model included the treatment group, type 2 diabetes mellitus randomization stratum, KCCQ scores at baseline, 4 months, and 8 months, and a categorical variable representing the number of investigator reported HF events (0, 1, ≥2 events) in the interval from randomization to 4 months, and in the interval from 4 to 8 months. Patients who died were counted as not improved in the analysis of improvement, or deteriorated in the analysis of deterioration. Patients with a baseline KCCQ score too high for them to experience an improvement according to a certain threshold (eg, baseline score ≥95 points for the 5-point threshold) were defined as improved if their score remained high (ie, ≥95 points) at 8 months. Similarly, patients with at KCCQ score at baseline too low for them to experience a deterioration were defined as deteriorated if their score remained low at 8 months. Number needed to treat (NNT) with their corresponding 95% CI were calculated using the method described by Bender.^13^ All analyses were conducted using STATA version 15.1 (College Station, TX) and SAS version 9.4 (SAS Institute, Cary, NC). A *P* value of 0.05 was considered statistically significant.

## Results

### Patient Characteristics

Overall, 4443 patients (93.7% of the overall trial population) had available KCCQ data at baseline. Baseline characteristics of patients with recorded vs missing KCCQ-TSS at randomization are presented in Table I in the online-only Data Supplement. There were a few modest differences between those with and without available KCCQ-TSS at baseline, although most clinical characteristics were similar. Notably, patients randomized to dapagliflozin versus placebo were equally distributed among those with recorded and missing KCCQ-TSS at baseline. Importantly, there was also no difference in clinical outcomes between patients that had KCCQ-TSS recorded versus missing at randomization (Table II in the online-only Data Supplement). Of these, 4141 patients (89.7% of surviving patients) had KCCQ evaluated at 4 months (130 missing KCCQ data due to death, 473 missing for reasons other than death); and 3955 (88.3% of surviving patients) had KCCQ evaluated at 8 months (257 missing KCCQ data due to death, 532 missing KCCQ data for reasons other than death). The proportions of patients with missing KCCQ values were similar in the dapagliflozin and placebo groups at 4 months and 8 months (89.9% vs 89.6%; and 88.7% vs 87.6%, respectively). The median KCCQ-TSS was 77.1 (interquartile range, 58.3–91.7). The number and proportion of patients in the KCCQ-TSS tertiles are shown in Table. Compared with participants with higher KCCQ-TSS scores at baseline, those with lower scores were younger, more often women, white, and enrolled in Europe and the Americas (and less likely to be enrolled in Asia—an observation also made in previous HFrEF global trials^14^). They also had a higher body mass index and natriuretic peptide levels; and a lower estimated glomerular filtration rate (Table); and were more likely to be in NYHA functional class III/IV than in class II, and to have type 2 diabetes mellitus and atrial fibrillation. With respect to background HF medications, patients with lower baseline KCCQ-TSS were more frequently treated with mineralocorticoid receptor antagonists and diuretics. Baseline use of angiotensin receptor neprilysin inhibitor was generally low but similar across age groups. The proportion of patients treated with implantable cardiac devices was generally comparable across the KCCQ-TSS subgroups.

**Table.**
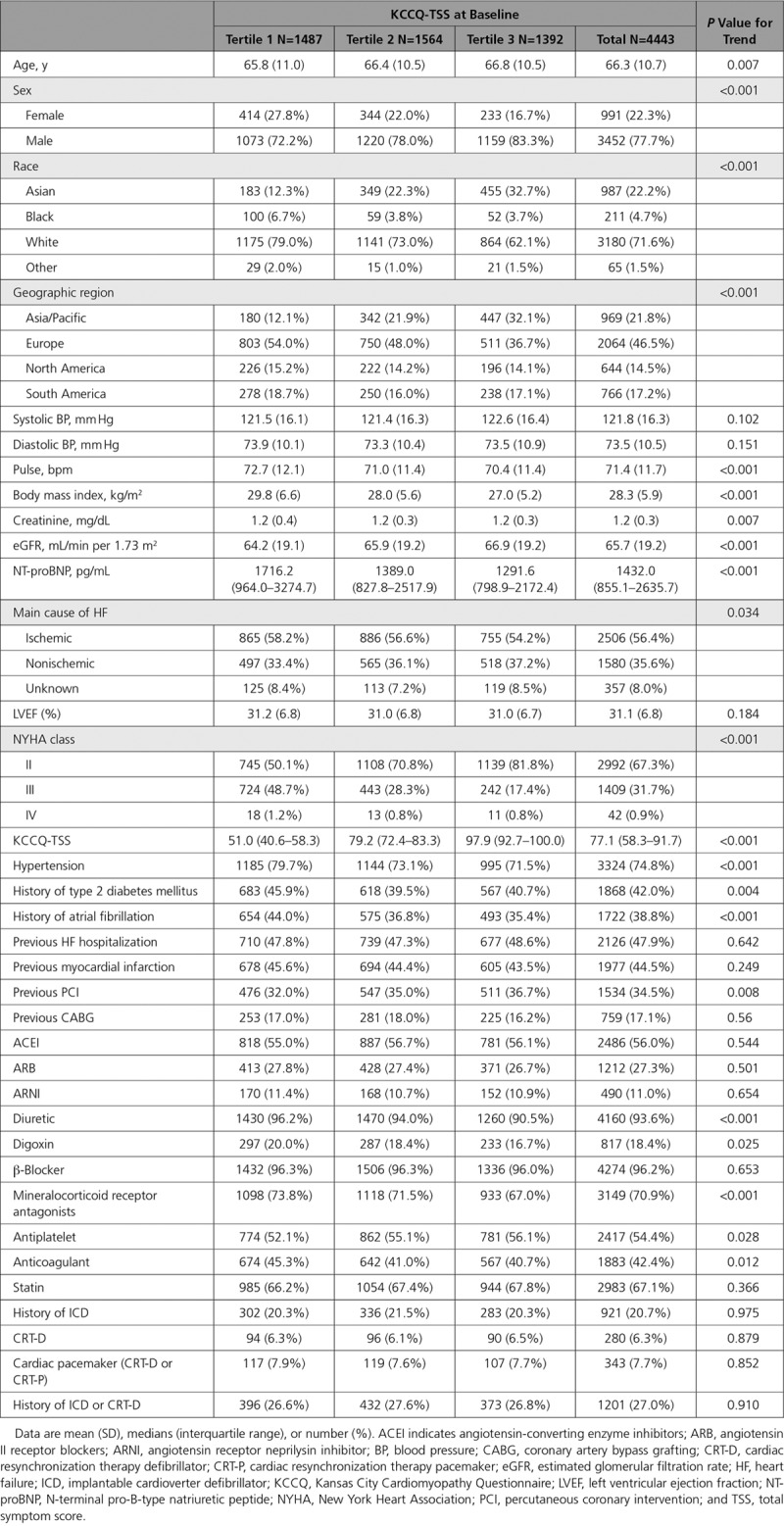
Baseline Characteristics of the KCCQ Study Population

### Clinical Outcomes

**Figure 1.**
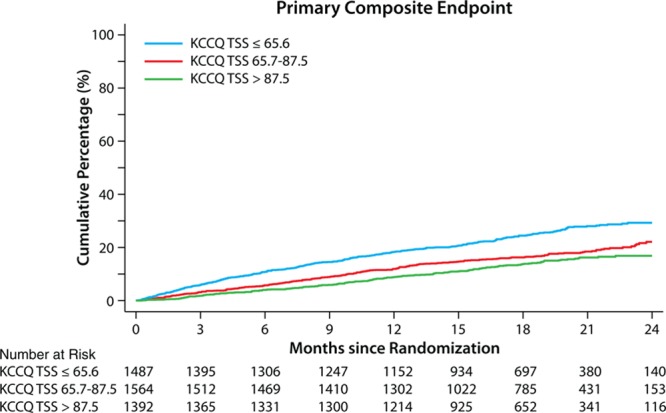
**Time to first event of cardiovascular death or worsening heart failure according to KCCQ-TSS tertile at randomization.** Tertile >87.5: referent; tertile 65.7–87.5: HR, 1.30 (95% CI, 1.08–1.56), *P*=0.006; tertile ≤65.6: HR, 1.93 (95% CI, 1.62–2.30), *P*<0.001. HR indicates hazard ratio; KCCQ, Kansas City Cardiomyopathy Questionnaire; and TSS, total symptom score.

Patients with lower baseline KCCQ-TSS experienced higher rates of cardiovascular death or worsening HF (25.0%, 17.3%, and 13.6% in patients across KCCQ-TSS tertiles of ≤65.6, 65.7–87.5, and >87.5, respectively; *P*<0.001). In the Cox proportional hazards models, patients with lower baseline KCCQ-TSS had a higher risk of cardiovascular death or worsening HF (tertile >87.5: referent; tertile 65.7–87.5: HR, 1.30 [95% CI, 1.08–1.56], *P*=0.006; tertile ≤65.6: HR, 1.93 [95% CI, 1.62–2.30], *P*<0.001; Figure 1).

**Figure 2.**
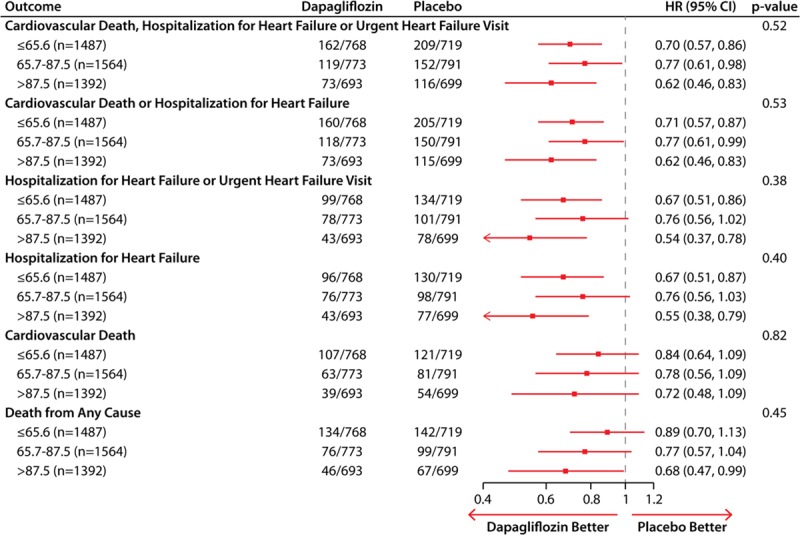
**Effects of dapagliflozin as compared with placebo on the clinical events across the tertiles of KCCQ-TSS at baseline.** HR indicates hazard ratio; KCCQ, Kansas City Cardiomyopathy Questionnaire; and TSS, total symptom score.

The effects of dapagliflozin on the range of clinical outcomes are summarized in Figure 2. Dapagliflozin reduced the primary outcome of cardiovascular death or worsening HF across the entire range of KKCQ-TSS, with no evidence of treatment effect heterogeneity; and with patients in each tertile experiencing a statistically significant benefit (HR [95% CIs] from lowest to highest tertile: HR, 0.70 [95% CI, 0.57–0.86]; HR, 0.77 [95% CI, 0.61–0.98]; and HR, 0.62 [95% CI, 0.46–0.83], respectively; *P* for heterogeneity = 0.52). Similar results were observed for cardiovascular death or hospitalization for HF; worsening HF events; HF hospitalizations; cardiovascular death; and all-cause death (Figure 2; all *P* values for heterogeneity nonsignificant).

### Health Status Outcomes

**Figure 3.**
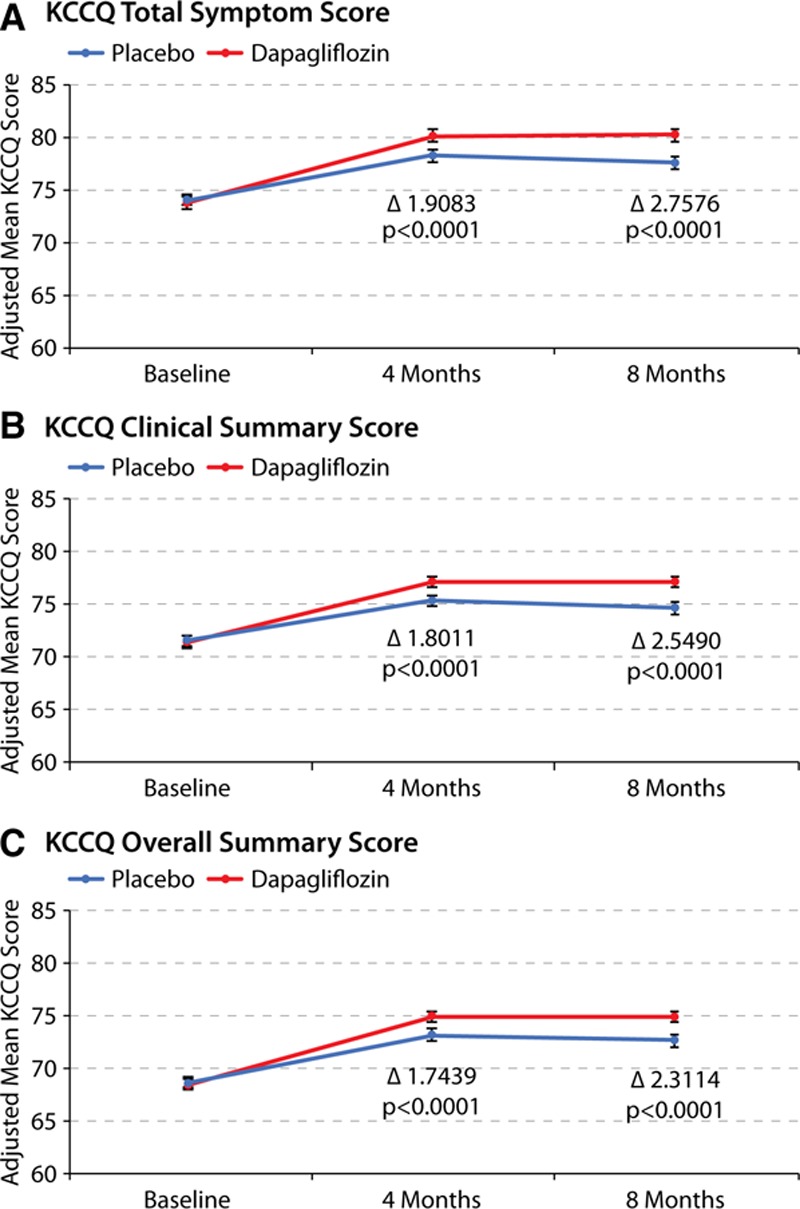
**Effects of dapagliflozin compared with placebo on KCCQ scores over 8 months.** Effects of dapagliflozin as compared with placebo on mean (**A**) KCCQ-TSS, (**B**) KCCQ-CSS, and (**C**) KCCQ-OSS over 8 months of treatment. Analysis includes those patients that are alive at the time of the KCCQ assessment (ie, 4 months and 8 months). CSS indicates clinical summary score; KCCQ, Kansas City Cardiomyopathy Questionnaire; OSS, overall summary score; and TSS, total symptom score.

The mean changes in KCCQ-TSS, CSS, and OSS over time are presented in Figure 3A through 3C, respectively. Patients treated with dapagliflozin had a modest but significant improvement in mean KCCQ-TSS, CSS, and OSS at 4 months (1.9, 1.8, and 1.7 points higher than placebo, respectively; *P*<0.0001 for all). These differences between dapagliflozin and placebo were amplified over time, with the corresponding mean differences at 8 months being 2.8, 2.5, and 2.3 points higher in favor of dapagliflozin versus placebo (*P*<0.0001 for all).

**Figure 4.**
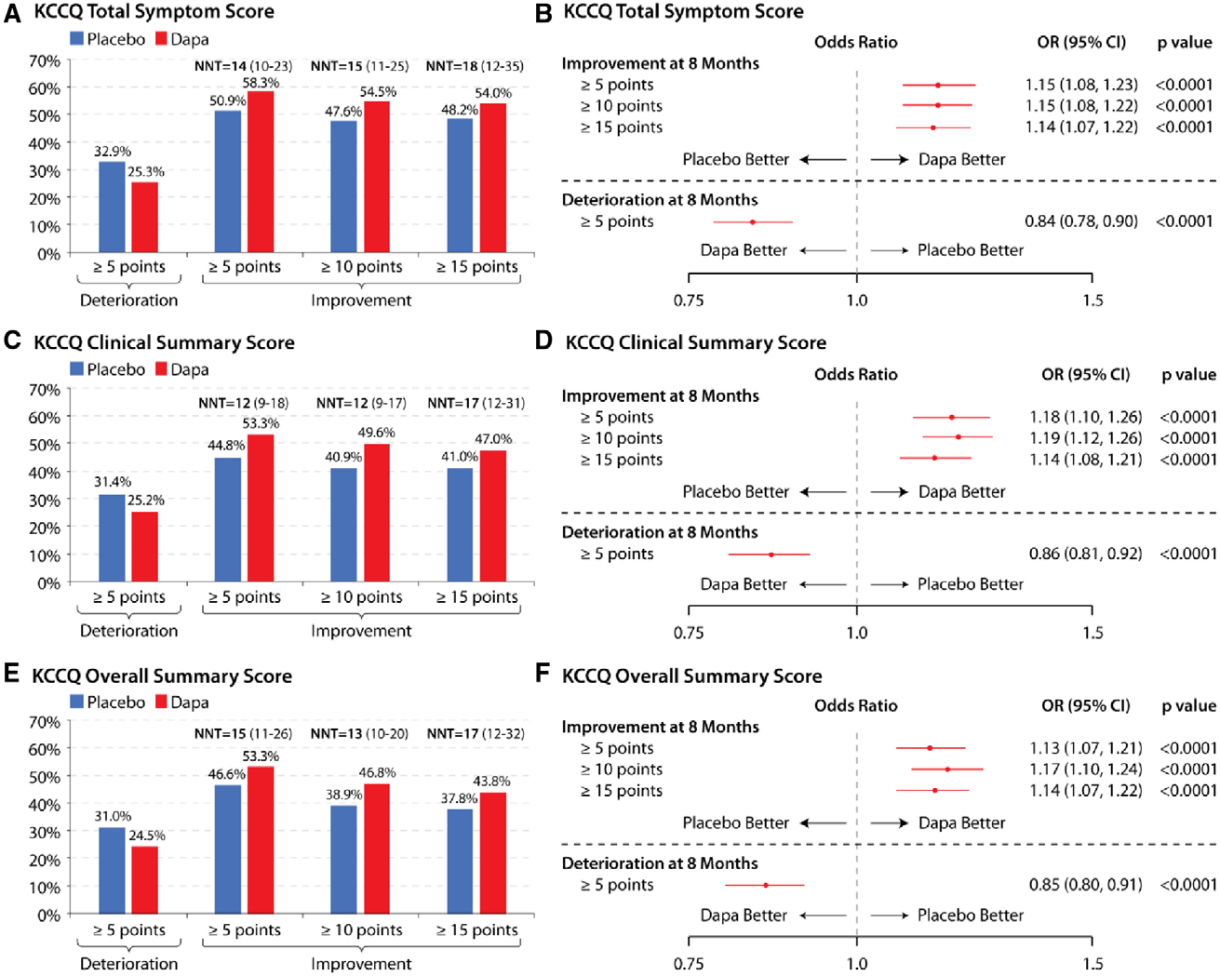
**Responder analyses of clinically meaningful change in KCCQ at 8 months with dapagliflozin versus placebo.** Responder analyses of clinically meaningful changes in (**A** and **B**) KCCQ-TSS, (**C** and **D**) KCCQ-CSS, and (**E** and **F**) KCCQ-OSS with dapagliflozin vs placebo at 8 months. Deaths are treated as not improved or as deteriorated (for the improvement and deterioration calculations, respectively). CSS indicates clinical summary score; Dapa, dapagliflozin; KCCQ, Kansas City Cardiomyopathy Questionnaire; NNT, number needed to treat (numbers in parentheses represent 95% CIs); OR, odds ratio; OSS, overall summary score; and TSS, total symptom score.

The results of the responder analysis are shown in Figure 4A through 4D. Fewer patients treated with dapagliflozin had a clinically significant deterioration (≥5 point decline in KCCQ-TSS [25.3% versus 32.9%; OR, 0.84 {95% CI, 0.78–0.90}, *P*<0.0001]); and more patients treated with dapagliflozin had at least small (58.3% versus 50.9%), moderate (54.5% versus 47.6%), and large (54.0% versus 48.2%) improvements (corresponding OR [95% CI]: OR, 1.15 [95% CI, 1.08–1.23]; OR, 1.15 [95% CI, 1.08–1.22]; OR, 1.14 [95% CI, 1.07–1.22]; NNT [95% CI]: NNT, 14 [95% CI, 10–23]; NNT, 15 [95% CI, 11–25]; and NNT, 18 [95% CI, 12–35], respectively; *P*<0.0001 for all; Figure 4A and 4B). The findings were similar for KCCQ-CSS and OSS (Figure 4C through 4F).

## Discussion

In this prospective study, which evaluated prespecified assessments of health status using KCCQ in the DAPA-HF trial, we observed that treatment with dapagliflozin reduced the risk of all key clinical events, including the primary composite end point of cardiovascular death or worsening HF, and its components, to a similar extent across the entire range of KCCQ at baseline, indicating that the beneficial effects of dapagliflozin on HF outcomes are independent of the health status impairment at baseline. Furthermore, dapagliflozin significantly improved KCCQ-TSS, CSS, and OSS (which collectively encompass symptoms, physical function, quality of life, and social function), and these effects were amplified over time. Finally, significantly fewer patients treated with dapagliflozin experienced clinically meaningful deterioration, and significantly more experienced at least small, moderate, and large clinically meaningful improvements in health status. These effects were substantial, with NNT ranging between 12 and 18 after just 8 months of treatment.

Our results have several important implications. First, our analyses of the clinical outcomes across the subgroups of baseline KCCQ-TSS show no evidence of heterogeneity in the benefit of dapagliflozin by the magnitude of symptomatic impairment at baseline. Previously reported prespecified subgroup analyses of the primary end point (cardiovascular death or worsening HF) suggested that the benefit of dapagliflozin may be more pronounced in patients with NYHA class II versus class III to IV. ^6^ However, NYHA class, although prognostically important, represents a more subjective, arbitrary, and non–patient-centric assessment of symptom burden; and considering this report, the observation from the previous NYHA class subgroup analysis was likely a chance finding.

Second, our findings substantially expand on the previously reported effects of dapagliflozin on health status, as measured by KCCQ, in patients with HFrEF. In the DEFINE-HF trial (Dapagliflozin Effects on Biomarkers, Symptoms, and Functional Status in Patients With Heart Failure With Reduced Ejection Fraction), a modestly sized randomized, placebo-controlled trial performed across 26 sites in the United States, dapagliflozin was also shown to have favorable effects on several domains of KCCQ—with slightly greater mean differences in favor of dapagliflozin versus placebo (ie, 4.8 points for KCCQ-TSS) than those observed in the DAPA-HF trial, but comparable responder analyses and NNT (ie, NNT of 10 for 5-point or greater improvement in KCCQ-OSS), after just 12 weeks of treatment.^15^ Our findings confirm these beneficial effects on symptoms, function, and quality of life in a much larger, global trial, with a longer duration of follow-up and the ability to assess the effects of dapagliflozin on clinical outcomes across the range of baseline KCCQ. Collectively, these complimentary findings from both the DEFINE-HF and DAPA-HF trials indicate that dapagliflozin significantly improves HF-related health status, as measured by KCCQ, with the benefits emerging early and being sustained long-term.

Third, the magnitude of the improvement in KCCQ that we observed with dapagliflozin versus placebo in the DAPA-HF trial compares favorably with other efficacious therapies for HFrEF. As an example, in the SHIFT trial (Systolic Heart Failure Treatment With the *If* Inhibitor Ivabradine Trial),^16^ ivabradine demonstrated a 2.4-point mean improvement in KCCQ-OSS, and a 1.8-point mean improvement in KCCQ-CSS after 12 months of treatment. In the PARADIGM-HF study (Prospective Comparison of ARNI [Angiotensin Receptor Neprilysin Inhibitor] With ACEI [Angiotensin-Converting-Enzyme Inhibitor] to Determine Impact on Global Mortality and Morbidity in Heart Failure),^17^ sacubitril-valsartan demonstrated 1.3- and 0.9-point improvements in KCCQ-OSS and KCCQ-CSS, respectively, over enalapril after 8 months of treatment (although true baseline measurement of KCCQ was not done, which complicates the interpretation of these data). In the HF-ACTION trial (Heart Failure: A Controlled Trial Investigating Outcomes of Exercise Training), exercise therapy in HFrEF produced a 1.9-point improvement in KCCQ-OSS.^18^ In the MADIT-CRT study (Multicenter Automatic Defibrillator Implantation Trial With Cardiac Resynchronization Therapy)^19^ in patients with HFrEF and prolonged QRS interval, treatment with cardiac resynchronization therapy resulted in 2.0-, 2.0-, and 2.4-point improvements in KCCQ-TSS, CSS, and OSS, respectively, in patients with left bundle branch block, and no significant improvements in KCCQ among patients without left bundle branch block. It should be noted, however, that comparisons of mean between-group differences in KCCQ do not adequately reflect clinically important changes in individual patients (because the effects are averaged across large populations). Therefore, from a clinical standpoint, the responder analyses that calculate the proportions of individual patients who experience a clinically meaningful change (deterioration or improvement in KCCQ) are more informative. Although few responder analyses had been done previously, the magnitude of benefit (including NNT) observed with dapagliflozin in the responder analyses of DAPA-HF also compare very favorably with previously observed results.^16,17^ It should further be noted that the NNT for clinically meaningful improvements in KCCQ observed in the DAPA-HF trial should be interpreted in the context of comparing dapagliflozin-treated patients with those that received placebo (who also experienced an improvement in health status, consistent with a sizable “placebo effect,” seen in our study, in the DEFINE-HF trial with dapagliflozin, and in placebo-controlled trials of other agents in HFrEF).^16^ Given the importance of reducing symptom burden and functional limitations and improving the quality of life—a key goal of HF management endorsed by the practice guidelines and regulators—our findings provide further support for dapagliflozin as a new treatment option for patients with HFrEF.

The results of our study should be considered in the context of several potential limitations. Although KCCQ was a predefined secondary end point, and prospective assessments of health status were specified in protocol, the evaluation of clinical outcomes (such as cardiovascular death or worsening HF) by tertiles of baseline KCCQ-TSS was done post hoc. The number of black patients was relatively small, although similar to other global HFrEF trials. KCCQ data were missing in a small proportion of patients. As in other trials, the prespecified inclusion and exclusion criteria will have reduced enrollment of hospitalized and other very high-risk patients. These limitations may affect the generalizability of our results.

### Conclusions

In the DAPA-HF trial, treatment with dapagliflozin reduced death and HF hospitalizations across the range of baseline KCCQ values, and improved symptom burden, functional status, and quality of life in patients with HFrEF. Furthermore, dapagliflozin significantly increased the proportion of patients experiencing small, moderate, and large improvements in health status; these effects were clinically important.

## Acknowledgments

The authors acknowledge Róisin O’Connor (InScience Communications, Springer Healthcare) for administrative assistance with manuscript submission.

## Sources of Funding

The DAPA-HF trial was funded by AstraZeneca. Administrative support in manuscript submission was also funded by AstraZeneca. Prof McMurray is supported by a British Heart Foundation Centre of Research Excellence Grant RE/18/6/34217.

## Disclosures

Dr Kosiborod has received grants, honoraria and other research support from AstraZeneca, grants and honoraria from Boehringer Ingelheim, and honoraria from Sanofi, Amgen, NovoNordisk, Merck (Diabetes), Eisai, Janssen, Bayer, GlaxoSmithKline, Glytec, Intarcia, Novartis, Applied Therapeutics, Amarin, and Eli Lilly. Dr Jhund’s employer (University of Glasgow) is paid by AstraZeneca for involvement in the DAPA-HF trial. He has also received consulting, advisory board, and speaker’s fees from Novartis, advisory board fees from Cytokinetics, and a grant from Boehringer Inhelheim. Dr Docherty’s employer (University of Glasgow) is paid by AstraZeneca for involvement in the DAPA-HF trial. He has also received a grant from Novartis. Dr Diez received personal fees from AstraZeneca for serving as National Lead Investigator for DAPA-HF. Dr Petrie’s institute is paid by AstraZeneca for his services as National Lead Investigator for DAPA-HF. He has also received personal fees from AstraZeneca, Novartis, and Lilly for being a speaker, from Takeda, Bayer, and Alnylam for being part of Endpoint Committees, from Napp for advisory board participation, from NovoNordisk for advisory board, speaker, and Endpoint Committee participation, and from Boehringer Ingelheim for advisory board and Endpoint Committee participation, and has also received a grant from Boehringer Ingelheim for research/clinical trial funding. Dr Verma received financial support from AstraZeneca for the conduct of DAPA-HF at his institute. He has also received grants and personal fees for speaker honoraria and advisory board participation from AstraZeneca, Bayer, Boehringer Ingelheim, Janssen, and Merck. He has received grants and personal fees for advisory board participation from Amgen, grants from Bristol-Myers Squibb, personal fees for speaker honoraria and advisory board participation from Eli Lilly, Novo Nordisk and Sanofi, and personal fees for speaker honoraria from EOCI Pharmacomm Ltd, Novartis, Sun Pharmaceuticals and Toronto Knowledge Translation Working Group. Dr Nicolau has received a grant from AstraZeneca for being National Lead Investigator for DAPA-HF and for other studies, a grant and personal fees from Bayer for being a Principal Investigator, grants from Sanofi for being a National Lead Investigator and a Principal Investigator, grants from Bristol Myers Squibb, NovoNordisk, and Novartis for being a Principal Investigator, grants from CLS Behring, Dalcor, Janssen, and Vifor for being a National Lead Investigator, personal fees from Amgen and Novartis for being a consultant, from Daiichi-Sankyo for being a speaker, and from Sanofi and Servier for advisory board participation. Dr Merkely has received grants and personal fees for acting as a speaker for Abbott and Medtronic, and personal fees for acting as a speaker for AstraZeneca, Sanofi Aventis, Servier, and Biotronik. Dr Kitakaze has received personal fees from AstraZeneca for lectures on DAPA-HF, and has received research grants and personal fees for lecturing from Asteras, Sanofi, Pfizer, Ono, Novartis, and Tanabe-Mitsubishi, has received personal fees for lecturing from Daiichi-Sankyo, Bayer, Boehinger, Kowa, Sawai, MSD, Shinogi, Kureha, Taisho-Toyama, Takeda, and Toa Eiyo, and has received personal fees for manuscripts from Japan Medical Data. Dr DeMets has received personal fees from Frontier Science and DL DeMets Consulting for serving as a consultant, is an owner of DL DeMets Consulting, and has received personal fees from Actelion, Population Health Research Institute, Duke Clinical Research Institute, Bristol Myers Squibb, Medtronic, Boston Scientific, GlaxoSmithKline, and Merck for participating in Consultant Data Monitoring Committees, from the National Institute of Allergy and Infectious Diseases (National Institutes of Health) for participating in the Ebola Data Monitoring Committee, and from National Heart, Lung, and Blood Institute (National Institutes of Health) for participating in the Ischemia Trial Data Monitoring Committee. Dr Inzucchi has received personal fees from AstraZeneca for being an Executive Committee member and an advisor and nonfinancial support for travel costs, personal fees from Boehringer Ingelheim for being a speaker/consultant and a Clinical Trial Publications Committee member and nonfinancial support for travel costs and payments in kind (medical writing assistance in manuscript preparation), personal fees from Sanofi/Lexicon for being a Clinical Trial Steering Committee member and nonfinancial support for travel costs, personal fees from Merck for being a speaker/consultant and nonfinancial support for travel costs, personal fees from Zafgen for being an advisor, and personal fees from VTV Therapeutics and Abbott/Alere for being an advisor and nonfinancial support for travel costs. Dr Køber is an executive committee member for the DAPA-HF study, payment from which will be administered by Rigshospitalet University Hospital, and has received personal fees from Novartis and Bristol Myers Squib for acting as a speaker. Dr Martinez has received personal fees from AstraZeneca as honoraria for being an Executive Committee Member for DAPA-HF. Dr Ponikowski was an investigator in the DAPA-HF trial and received personal fees from AstraZeneca for lectures and consultancy related to the trial. He has also participated in clinical trials and received research grants to his institute and personal fees for speakers bureau and consultancy from Vifor Pharma. He has participated in clinical trials and received personal fees for consultancy and speakers bureaus from Boehringer Ingelheim, Bayer, Bristol Myers Squibb, Cibiem, Novartis, and RenalGuard, personal fees for speakers bureaus and consultancy from Servier and Respicardia, personal fees for speakers bureaus from Berlin-Chemie, and personal fees for lectures from Pfizer. Dr Sabatine has received a research grant to the TIMI Study Group (Thombolysis in Myocardial Infarction) at his institution from AstraZeneca for his participation in DAPA-HF, has received a research grant to the TIMI Study Group at his institution and personal fees for consulting from Amgen, AstraZeneca, Intarcia, Janssen Research and Development, Medicines Company, MedImmune, Merck, and Novartis, research grant to the TIMI Study Group at his institution from Bayer, Daiichi-Sankyo, Eisai, GlaxoSmithKline, Pfizer, Poxel, Quark Pharmaceuticals, and Takeda, and personal fees for consulting from Anthos Therapeutics, Bristol Myers Squibb, CVS Caremark, DalCor, Dyrnamix, Esperion, IFM Therapeutics, and Ionis. He is also a member of the TIMI Study Group, which has received institutional research grant support through his institute from Abbot, Aralez, Roche, and Zora Biosciences. Dr Solomon has received a grant to his institution from AstraZeneca for being an Executive Committee Member for DAPA-HF and has received grants to his institute and personal fees for consulting from Alnylam, Amgen, AstraZeneca, Bristol Myers Squibb, Gilead, GlaxoSmithKline, MyoKardia, Novartis, Theracos, and Bayer, has received grants to his institute from Bellerophon, Celladon, Ionis, Lone Star Heart, Mesoblast, National Heart, Lung, and Blood Institute (National Institutes of Health), and Sanofi Pasteur, and personal fees for consulting from Akros, Corvia, Ironwood, Merck, Roche, Takeda, Quantum Genomics, and AoBiome. Dr Bengtsson reports personal fees from AstraZeneca outside the submitted work. Drs Lindholm and Sjostrand are employees of AstraZeneca. Drs Niklasson and Langlidke are employees and shareholders of AstraZeneca. Dr McMurray’s employer (University of Glasgow) is paid by AstraZeneca for his role as Principal Investigator in the DAPA-HF trial and Co-Principal Investigator in the DELIVER trial (Dapagliflozin Evaluation to Improve the Lives of Patients With Preserved Ejection Fraction Heart Failure). He has also received nonfinancial support for travel and accommodation from AstraZeneca for meetings related to these trials. Dr McMurray’s employer has been paid by Cardiorentis for his time spent as Steering Committee member and Endpoint Committee Chair and related meetings, and he has received nonfinancial support for travel and accommodation for some related meetings. Dr McMurray’s employer has been paid by Amgen, Oxford University/Bayer, Abbvie, and Bristol Myers Squibb for his time spent as a Steering Committee member and related meetings, and he has received nonfinancial support for travel and accommodation for some related meetings. Dr McMurray’s employer has been paid by Kings College Hospital/Kidney Research UK/Vifor-Fresenius for his time spent as a Steering Committee member and for running an Endpoint Adjudication Committee and related meetings, and he has received nonfinancial support for travel and accommodation for some related meetings. Dr McMurray’s employer has been paid by Theracos for his time spent as Principal Investigator and related meetings, and he has received nonfinancial support for travel and accommodation for some related meetings. Dr McMurray’s employer has been paid by Pfizer and Merck for his time spent on the Data Safety Monitoring Committee and related meetings. Dr McMurray’s employer has been paid by Novartis for his time spent as Executive/Steering Committee member, Co-Principal Investigator, and Advisory Board member, and he has received nonfinancial support for travel and accommodation for some related meetings/presentations. Dr McMurray’s employer has been paid by Bayer for his participation as a Steering Committee member, by DalCor Pharmaceuticals for his participation as a Steering Committee member (and related meetings), and by Bristol Myers Squibb for his participation as a Steering Committee member (and related meetings). Dr McMurray’s employer has been paid by GlaxoSmithKline for his participation as a Steering Committee member and Co-Principal Investigator, and he has received nonfinancial support for travel and accommodation for some related meetings. All payments for meetings-related travel and accommodation were made through a Consultancy with University of Glasgow, and Dr McMurray has not received personal payments in relation to any trials/drugs.

## Supplementary Material
