# Supplementary material for: Effects of Dapagliflozin on Symptoms, Function, and Quality of Life in Patients With Heart Failure and Reduced Ejection Fraction: Results From the DAPA-HF Trial
Source: Circulation. 2019 Nov 17;141(2):90–9. doi: 10.1161/CIRCULATIONAHA.119.044138 (PMC6964869; doi:10.1161/CIRCULATIONAHA.119.044138)
Supplement: Supplementary file 1 [file cir-141-90-s001.pdf]

SUPPLEMENTAL MATERIAL

Supplemental Tables

Supplemental Table 1: Baseline characteristics in patients with recorded vs missing KCCQ-TSS at randomization

	Baseline KCCQ recorded N=4,443	Missing baseline KCCQ data N=301	p-value
Randomized to dapagliflozin	2,234 (50.3%)	139 (46.2%)	0.17
Age – yr	66.3±10.7	66.8±13.4	0.44
Female sex – no. (%)	991 (22.3)	118 (39.2)	<0.001
Race – no. (%)			<0.001
Asian	987 (22.2)	129 (42.9)	
Black Or African American	211 (4.7)	15 (5.0)	
Other	65 (1.5)	4 (1.3)	
White	3,180 (71.6)	153 (50.8)	
Region – no. (%)			<0.001
North America	644 (14.5)	33 (11.0)	
South America	766 (17.2)	51 (16.9)	
Europe	2,064 (46.5)	90 (29.9)	
Asia/Pacific	969 (21.8)	127 (42.2)	
NYHA functional classification – no. (%)			0.39
II	2,992 (67.3)	211 (70.1)	
III	1,409 (31.7)	89 (29.6)	
IV	42 (0.9)	1 (0.3)	
Heart rate – beats/min	71.4±11.7	73.5±12.1	0.003
Systolic Blood Pressure – mm Hg	121.8±16.3	121.8±17.0	0.96
Left ventricular ejection fraction – %	31.1±6.8	30.7±6.9	0.38
Median NT-proBNP (IQR) – pg/ml	1432.0 (855.1–2635.7)	1493.3 (891.6–2735.7)	0.34
Baseline HbA1c (%)	6.5±1.3	6.5±1.5	0.86
Principal cause of heart failure – no. (%)			0.93
Ischemic	2,506 (56.4)	168 (55.8)	
Nonischemic	1,580 (35.6)	107 (35.5)	
Unknown	357 (8.0)	26 (8.6)	
Medical history – no. (%)			
Hospitalization for heart failure	2,126 (47.9)	125 (41.5)	0.034
Atrial fibrillation	1,722 (38.8)	96 (31.9)	0.018
Diabetes	1868 (42.0)	115 (38.2)	0.19
Estimated GFR			
eGFR – mL/min/1.73m^2	65.7±19.2	67.6±22.2	0.098
eGFR < 60 mL/min/1.73m^2	1,805 (40.6)	121 (40.2)	0.88
Device therapy – no. (%)			
Implantable cardioverter-defibrillator	1,201 (27.0)	41 (13.6)	<0.001
Cardiac resynchronization therapy	343 (7.7)	11 (3.7)	0.009
Heart failure medication – no. (%)			
Diuretic	4,160 (93.6)	273 (90.7)	0.047
ACE inhibitor	2,486 (56.0)	175 (58.1)	0.46
Angiotensin receptor blocker	1,212 (27.3)	95 (31.6)	0.11
Sacubitril-valsartan	490 (11.0)	18 (6.0)	0.006
Beta-blocker	4,274 (96.2)	284 (94.4)	0.11
Mineralocorticoid receptor antagonist	3,149 (70.9)	221 (73.4)	0.35
Digoxin	817 (18.4)	70 (23.3)	0.036
Diabetes medications* – no. (%)			
Metformin	963 (50.8)	67 (58.3)	0.81
Sulfonylurea	406 (21.7)	32 (27.8)	0.40
DPP4-inhibitor	298 (16.0)	12 (10.4)	0.065
GLP-1 receptor agonist	20 (1.1)	1 (0.9)	0.77
Insulin	510 (27.3)	30 (26.1)	0.42

*Glucose-lowering medications are listed only for the patients who had a history of diabetes at baseline; Data are mean ± standard deviation, median (interquartile range) or no. (%); ACE, angiotensin-converting enzyme;

DPP4; dipeptidyl peptidase-4; eGFR, estimated glomerular filtration rate; GLP-1, glucagon-line peptide-1; KCCQ, Kansas City Cardiomyopathy Questionnaire; NT-proBNP, N-terminal pro b-type natriuretic peptide; NYHA, New York Heart Association; TSS, total symptom score; Yr, year

Supplemental Table 2: Clinical outcomes by recorded versus missing KCCQ-TSS at randomization

	Baseline KCCQ data recorded	Baseline KCCQ data missing	p-value
**Cardiovascular death/HF Hosp/Urgent HF visit**	831 (18.7%)	57 (18.9%)	0.92
**Cardiovascular death/HF Hosp**	821 (18.5%)	56 (18.6%)	0.96
**HF Hosp/Urgent HF visit**	533 (12.0%)	30 (10.0%)	0.29
**HF Hosp**	520 (11.7%)	29 (9.6%)	0.28
**Cardiovascular death**	465 (10.5%)	35 (11.6%)	0.53
**All cause death**	564 (12.7%)	41 (13.6%)	0.64

Data are no. (%); HF, heart failure; Hosp, hospitalization; KCCQ, Kansas City Cardiomyopathy Questionnaire; TSS, total symptom score
